# Further evidence for a male-selective genetic association of synapse-associated protein 97 (*SAP97*) gene with schizophrenia

**DOI:** 10.1186/1744-9081-8-2

**Published:** 2012-01-06

**Authors:** Akihito Uezato, Junko Kimura-Sato, Naoki Yamamoto, Yoshimi Iijima, Hiroshi Kunugi, Toru Nishikawa

**Affiliations:** 1 Department of Psychiatry and Behavioral Sciences, Graduate School of Medical and Dental Sciences, Tokyo Medical and Dental University, 1-5-45 Yushima, Bunkyo-ku, Tokyo 113-8519, Japan; 2 Department of Sleep Modulatory Medicine, Graduate School of Medical and Dental Sciences, Tokyo Medical and Dental University, 1-5-45 Yushima, Bunkyo-ku, Tokyo 113-8510, Japan; 3 Department of Psychiatry and Behavioral Neurobiology, University of Alabama at Birmingham, 1530 3rd Ave S, Birmingham, AL 35294, USA; 4 Department of Mental Disorder Research, National Institute of Neuroscience, National Center of Neurology and Psychiatry, 4-1-1, Ogawahigashimachi, Kodaira, Tokyo 187-8502, Japan

**Keywords:** Schizophrenia, genetic association, single nucleotide polymorphisms, synapse-associated protein, scaffolding protein, SAP97, gender selective, glutamate

## Abstract

**Background:**

The synapse-associated protein 97 gene (*SAP97*) encodes a regulatory scaffold protein for the localization of L-alpha-amino-3-hydroxy-5-methyl-4-isoxazole propionic acid (AMPA), kainate and N-methyl-D-aspartate (NMDA) type glutamate receptors. We have recently demonstrated nominally significant associations between *SAP97 *gene and schizophrenia among Japanese males. The present study aimed to replicate these findings using an independent and larger sample.

**Methods:**

We investigated seven *SAP97 *single nucleotide polymorphisms (SNPs) that displayed a significant association with schizophrenia in our preceding study in an independent Japanese population consisting of a total of 393 unrelated patients with schizophrenia (232 males and 161 females) and 393 unrelated control subjects (211 males and 182 females).

**Results:**

The SNP rs9843659 showed a significant genotypic association with male patients in a recessive model (p = 0.037). The analysis of the combined data from the current and prior studies also demonstrated a significant association of this SNP (p = 0.0039). The meta-analysis for the allele frequency covering the two studies yielded an odds ratio of 1.38.

**Conclusions:**

The present study replicated the previously reported male-selective genetic association between the *SAP97 *polymorphism and schizophrenia. These findings further support the possible involvement of the *SAP97 *gene variation in the susceptibility to schizophrenia in males and in the genetic basis for sex differences in the disorder.

## Background

Schizophrenia is a serious psychiatric disorder with a high prevalence of nearly 1% and a wide variety of mental dysfunctions that are only partially improved by the current antipsychotic drugs. It is well accepted that multiple susceptibility genes may be involved in the pathogenesis of schizophrenia [1], and the search for such genes has produced promising results [2]. To obtain a further insight into the genetic factors, we have investigated the possible association between schizophrenia and the genes that are related to the impaired N-methyl-D-aspartate (NMDA) receptor-mediated glutamate neurotransmission and the development-dependent onset of schizophrenia, because 1) NMDA receptor antagonists, such as phencyclidine (PCP) and ketamine, cause the psychotic symptoms indistinguishable from those of schizophrenia [3,4], 2) the onset of schizophrenia and the above psychotomimetic effects typically occur after puberty [5-9], and 3) in experimental animals, the adult type PCP- and dizocilpine (MK-801)-induced abnormal behavior as a model of schizophrenic symptoms is observed only after the weaning period [10-12]. Recently, we have shown that the synapse-associated protein 97 gene (*SAP97*), which encodes a regulatory scaffold protein for the localization of L-alpha-amino-3-hydroxy-5-methyl-4-isoxazole propionic acid (AMPA), kainate and NMDA type glutamate receptors [13], is upregulated in the adult but not in the infant rat after PCP administration [14]. Moreover, we have demonstrated nominally significant associations between *SAP97 *single nucleotide polymorphisms (SNPs) and schizophrenia among males [15]. In the present study, we therefore aimed to replicate the prior genetic association findings using an independent and larger sample.

## Methods

A total of 393 unrelated Japanese patients with schizophrenia (232 males, 41.9 ± 11.4 years, and 161 females, 40.7 ± 12.3 years) and 393 unrelated Japanese control subjects (211 males, 38.6 ± 10.9 years, and 182 females, 41.5 ± 12.9 years) were included in this study. All patients were diagnosed by well-trained psychiatrists, according to the Diagnostic and Statistical Manual of Mental Disorders, fourth edition (DSM-IV) criteria. All subjects resided in central Japan. The ethics committees of the Tokyo Medical and Dental University and National Center of Neurology and Psychiatry approved the present study. The control subjects were recruited through advertisements in free local information magazines and by our website announcement. They were interviewed using the Japanese version of the Mini-International Neuropsychiatric Interview (M.I.N.I.) [16,17] by a research psychiatrist, and those who had a current or past history of psychiatric disorders were not enrolled in the study. Participants were excluded if they had prior medical histories of a central nervous system disease or severe head injury, or if they met the criteria for substance abuse or dependence, or mental retardation. This study was conducted in accordance with the latest version of the Declaration of Helsinki.

We selected seven SNPs which displayed a significant association with schizophrenia in the previous study [15]. TaqMan SNP Genotyping Assay method (Applied Biosystems, Foster City, CA, USA) was used to genotype these SNPs as previously described [15]. The representative genotyping results were confirmed by the direct sequence and TA cloning methods.

A statistical analysis was performed using the PLINK 1.07 software package http://pngu.mgh.harvard.edu/purcell/plink/[18]. Deviation from the predicted Hardy-Weinberg equilibrium (HWE) was evaluated by the chi-square test. To compare the allele and genotype frequencies between cases and controls, Fisher's exact test was performed. The analysis for genotype frequencies was performed assuming dominant, recessive and co-dominant effects for each polymorphism. In the dominant model, both the major allele homozygote and the heterozygote were combined. In the recessive model, the variant was defined as only the major allele homozygote. The co-dominant model compared the major allele homozygote and heterozygote to the minor allele homozygote. A meta-analysis was performed using the package (rmeta) for R-software http://www.r-project.org. When there is no heterogeneity, the fixed-effects (Mantel-Haenszel) model was applied. Otherwise, the random-effects (DerSimonian-Laird) model was used.

## Results

The allele frequency and genotype distribution of each SNP are summarized in Table 1. The genotype distribution of all the SNPs showed no significant deviations from the HWE in the control or disease groups (p > 0.05).

**Table 1:** Allele frequencies and genotype distribution of SNPs on the SAP97 gene

	**Allele (1/2)**^**a**^				Genotype Frequency (%)			p value			Allele Frequency (%)		p value
					
		Sex	Group	HWE	1/1	1/2	2/2	Dom	Rec	Codom	1	2	Allelic
SNP I-2 rs382579	T/C	M	CTL	1	130 (61.6)	71 (33.6)	10 (4.7)	0.675	0.561	0.744	331 (78.4)	91 (21.6)	0.470
		
			SCZ	0.718	136 (58.6)	82 (35.3)	14 (6.0)				354 (76.3)	110 (23.7)	
		F	CTL	1	116 (63.7)	59 (32.4)	7 (3.8)	0.776	0.910	0.939	291 (79.9)	73 (20.1)	0.848
			SCZ	0.801	104 (64.6)	52 (32.3)	5 (3.1)				260 (80.7)	62 (19.3)	
		
		All	CTL	1	246 (62.6)	130 (33.1)	17 (4.3)	0.865	0.714	0.884	622 (79.1)	164 (20.9)	0.667
			SCZ	1	240 (61.1)	134 (34.1)	19 (4.8)				614 (78.1)	172 (21.9)	

SNP I-3 rs9843659	T/C	M	CTL	0.083	86 (40.8)	88 (41.7)	37 (17.5)	0.545	**0.037**	0.101	260 (61.6)	162 (38.4)	0.076
			SCZ	1	72 (31.0)	114 (49.1)	46 (19.8)				258 (55.6)	206 (44.4)	
		
		F	CTL	0.755	70 (38.5)	84 (46.2)	28 (15.4)	0.769	1	0.946	224 (61.5)	140 (38.5)	0.814
			SCZ	0.512	61 (37.9)	73 (45.3)	27 (16.8)				195 (60.6)	127 (39.4)	
		
		All	CTL	0.137	156 (39.7)	172 (43.8)	65 (16.5)	0.512	0.104	0.230	484 (61.6)	302 (38.4)	0.123
			SCZ	0.607	133 (33.8)	187 (47.6)	73 (18.6)				453 (57.6)	333 (42.4)	

SNP I-4 rs2122824	G/T	M	CTL	1	126 (59.7)	75 (35.5)	10 (4.7)	0.538	0.501	0.650	327 (77.5)	95 (22.5)	0.430
			SCZ	0.861	131 (56.5)	86 (37.1)	15 (6.5)				348 (75.0)	116 (25.0)	
		
		F	CTL	1	113 (62.1)	61 (33.5)	8 (4.4)	0.583	1	0.834	287 (78.8)	77 (21.2)	0.851
			SCZ	0.477	100 (62.1)	56 (34.8)	5 (3.1)				256 (79.5)	66 (20.5)	
		
		All	CTL	0.883	239 (60.8)	136 (34.6)	18 (4.6)	0.868	0.611	0.846	614 (78.1)	172 (21.9)	0.587
			SCZ	0.887	231 (58.8)	142 (36.1)	20 (5.1)				604 (76.8)	182 (23.2)	

SNP I-5 rs7650753	G/T	M	CTL	1	126 (59.7)	75 (35.5)	10 (4.7)	0.419	0.63	0.629	327 (77.5)	95 (22.5)	0.430
			SCZ	0.597	133 (57.3)	83 (35.8)	16 (6.9)				349 (75.2)	115 (24.8)	
		
		F	CTL	1	112 (61.5)	62 (34.1)	8 (4.4)	0.583	1	0.851	286 (78.6)	78 (21.4)	0.779
			SCZ	0.477	100 (62.1)	56 (34.8)	5 (3.1)				256 (79.5)	66 (20.5)	
		
		All	CTL	0.883	238 (60.6)	137 (34.9)	18 (4.6)	0.743	0.771	0.855	613 (78.0)	173 (22.0)	0.673
			SCZ	1	233 (59.3)	139 (35.4)	21 (5.3)				605 (77.0)	181 (23.0)	

SNP I-10 rs7638423	A/G	M	CTL	0.123	98 (46.4)	84 (39.8)	29 (13.7)	0.892	0.504	0.773	280 (66.4)	142 (33.6)	0.572
			SCZ	0.316	100 (43.1)	99 (42.7)	33 (14.2)				299 (64.4)	165 (35.6)	
		
		F	CTL	0.610	86 (47.3)	76 (41.8)	20 (11.0)	0.866	0.231	0.431	248 (68.1)	116 (31.9)	0.294
			SCZ	0.731	65 (40.4)	77 (47.8)	19 (11.8)				207 (64.3)	115 (35.7)	
		
		All	CTL	0.137	184 (46.8)	160 (40.7)	49 (12.5)	0.831	0.196	0.394	528 (67.2)	258 (32.8)	0.264
			SCZ	0.660	165 (42.0)	176 (44.8)	52 (13.2)				506 (64.4)	280 (35.6)	

SNP II-1 rs6805920	C/A	M	CTL	0.166	98 (46.4)	85 (40.3)	28 (13.3)	0.784	0.390	0.675	281 (66.6)	141 (33.4)	0.438
			SCZ	0.395	98 (42.2)	101 (43.5)	33 (14.2)				297 (64.0)	167 (36.0)	
		
		F	CTL	0.610	86 (47.3)	76 (41.8)	20 (11.0)	0.737	0.231	0.440	248 (68.1)	116 (31.9)	0.259
			SCZ	0.865	65 (40.4)	76 (47.2)	20 (12.4)				206 (64.0)	116 (36.0)	
		
		All	CTL	0.170	184 (46.8)	161 (41.0)	48 (12.2)	0.670	0.151	0.324	529 (67.3)	257 (32.7)	0.184
			SCZ	0.662	163 (41.5)	177 (45.0)	53 (13.5)				503 (64.0)	283 (36.0)	

SNP II-8 rs4916461	A/C	M	CTL	0.080	104 (49.3)	80 (37.9)	27 (12.8)	0.679	0.505	0.736	288 (68.2)	134 (31.8)	0.572
			SCZ	0.108	106 (45.7)	93 (40.1)	33 (14.2)				305 (65.7)	159 (34.3)	
		
		F	CTL	0.491	88 (48.4)	74 (40.7)	20 (11.0)	1	0.278	0.490	250 (68.7)	114 (31.3)	0.415
			SCZ	0.861	68 (42.2)	75 (46.6)	18 (11.2)				211 (65.5)	111 (34.5)	
		
		All	CTL	0.079	192 (48.9)	154 (39.2)	47 (12.0)	0.746	0.224	0.453	538 (68.4)	248 (31.6)	0.260
			SCZ	0.314	174 (44.3)	168 (42.7)	51 (13.0)				516 (65.6)	270 (34.4)	

SNP, single-nucleotide polymorphism; HWE, Hardy-Weinberg equilibrium; M, male; F, female; All, male and female combined; CTL, control; SCZ, schizophrenia; Dom, dominant model; Rec, Recessive model; Codom, Co-dominant model.

Bold values indicate significance level p < 0.05.

^a^Allele 1 and 2 represent the major and minor alleles of each SNP.

Regarding male patients with schizophrenia, we observed a genotypic association between schizophrenia and SNP I-3 (rs9843659) in the recessive model (p = 0.037). Because there was a significant difference in the mean age between patients and controls in the male group (p = 0.0016), we performed a logistic regression analysis in the recessive model to control the possible confounding effect of age. The analysis yielded a statistical significance (p = 0.028). The SNP I-3 association in the recessive model was also demonstrated in the combined data from the current and prior studies (p = 0.0039) (Table 2). The p value remained statistically significant after permutation testing (p = 0.0124, 10,000 permutations). SNP I-3 also showed an association in the co-dominant model as well as an allelic association (Table 2). The combined data further revealed allelic and genotypic associations in SNP II-1 and II-8 (Table 2). The meta-analysis for the allele frequency covering the current and prior studies yielded an odds ratio of 1.38 for SNP I-3, and 1.26 and 1.25 for SNP II-1 and II-8, respectively, in the fixed-effects model (Table 2).

**Table 2:** Combined analysis and meta-analysis from the current and prior studies

		**Combined analysis (p value)**^**a**^				**Meta-analysis**^**b**^
		
		Allelic	Dom	Rec	Codom	Odds Ratio (95% CI)
SNP I-2 rs382579	M	0.179	0.409	0.214	0.404	1.19 (0.92-1.53)
	F	0.494	1.000	0.475	0.751	1.11 (0.82-1.50)
	All	0.107	0.418	0.115	0.260	1.17 (0.97-1.42)

SNP I-3 rs9843659	M	**0.004**	0.121	**0.004**	**0.011**	**1.38 **(1.11-1.71)
	F	0.757	1.000	0.594	0.835	1.04 (0.82-1.33)
	All	**0.014**	0.210	**0.011**	**0.034**	**1.22 **(1.04-1.44)

SNP I-4 rs2122824	M	0.089	0.409	0.105	0.222	1.24 (0.96-1.59)
	F	0.657	0.527	0.427	0.436	1.07 (0.80-1.43)
	All	0.085	0.698	0.064	0.165	1.18 (0.98-1.42)

SNP I-5 rs7650753	M	0.115	0.327	0.164	0.275	1.22 (0.95-1.57)
	F	0.712	0.527	0.480	0.471	1.06 (0.79-1.42)
	All	0.114	0.607	0.104	0.246	1.18 (0.98-1.42)

SNP I-10 rs7638423	M	0.078	0.497	0.053	0.142	1.23 (0.98-1.53)
	F	0.303	0.783	0.100	0.150	1.06 (0.79-1.42)
	All	**0.038**	0.727	**0.010**	**0.028**	**1.20 **(1.01-1.41)

SNP II-1 rs6805920	M	**0.041**	0.497	**0.020**	0.061	**1.26 **(1.01-1.58)
	F	0.200	1.000	0.068	0.133	1.06 (0.79-1.42)
	All	**0.014**	0.602	**0.003**	**0.009**	**1.23 **(1.04-1.46)

SNP II-8 rs4916461	M	**0.046**	0.420	**0.037**	0.104	**1.25 **(1.00-1.57)
	F	0.269	0.888	0.120	0.215	1.16 (0.90-1.49)
	All	**0.021**	0.532	**0.008**	**0.027**	**1.22 **(1.03-1.44)

SNP, single-nucleotide polymorphism; M, male; F, female; All, male and female combined; Dom, dominant model; Rec, Recessive model; Codom, Co-dominant model; CI, confidence interval.

Bold values indicate significance level p < 0.05.

^a^Combined analysis was performed on the combined data from the current study and Sato et al.

^b^Meta-analysis was performed covering the current study and Sato et al. for allele frequency

In the present study no significant case-control allele or genotype differences was demonstrated when female patients with schizophrenia were compared to control females or when all subjects were analyzed (Table 1). However, for the combined data from the current and prior studies, we detected significant differences in the allele and genotype frequencies in the recessive and co-dominant models between all patients and controls for SNP I-3, I-10, II-1 and II-8. Likewise, the meta-analyses for the allele frequencies demonstrated statistically significant odds ratios for these SNPs (Table 2).

## Discussion

These findings add further support to our previous report in that the SNP rs9843659 in the *SAP97 *gene is genetically associated with male patients with schizophrenia. The allele C of this SNP conferred a risk for schizophrenia susceptibility in both studies. As replicating results of a genetic association study in independent samples is considered as a standard to demonstrate the relevance of a candidate gene [19], the results of the present study in conjunction with the previous study strongly support the view that *SAP97 *is a risk gene for male patients with schizophrenia.

In the most recent and largest genome-wide association studies (GWAS), the tag SNP rs1392705 on chromosome 3q29 closest to the SNP rs9843659 in the *SAP97 *gene demonstrated a nominal genome-wide significance (p = 8.45 × 10^-4^) [20-22]. Another genome-wide analysis of the copy-number variation (CNV) found a statistically significant excess of deletions in schizophrenia at 3q29 [23]. The telomeric breakpoint of the minimal deletion in this study was 4.7 kb from the transcriptional stop of the *SAP97 *gene. These findings also implicate *SAP97 *as a candidate gene for schizophrenia susceptibility.

The contributions of the rs9843659 polymorphism to the development of schizophrenia remains unexplained because 1) this SNP is found in the fourth large intron, but not in the exons that encode the amino acid sequences of the SAP97 protein including the functional domains and motifs, and 2) as previously described, bioinformatics tools we used failed to reveal any consensus sequences in the intron part that may play a role in the alternative promoter use or alternative splicing. The rs9843659 polymorphism could cause certain modifications in the regulation of transcription or translation of the *SAP97 *gene by an unidentified mechanism, for instance, a change in its higher-order genome structure. This type of a 'silent' SNP has indeed been observed to alternate substrate specificity in the Multidrug Resistance 1 (*MDR1*) gene [24].

From this view point, it is of interest to note that the expressional changes of the SAP97 proteins have been reported by two independent research groups in the dorsolateral prefrontal cortex of postmortem schizophrenic brains [25,26]. However, it is not totally excluded that these results might be due to long-term treatment with antipsychotic drugs [25,26] and another research group failed to observe any significant alteration in the SAP97 mRNA level in the cortical area [27]. Furthermore, animal experiments have revealed the mutual interactions between SAP97 and the NMDA receptor that is hypothesized to be dysfunctional in schizophrenia brains. Thus, the *SAP97 *gene knockdown reduced the surface expression of GluR1 and GluR2 and inhibited both the AMPA and NMDA excitatory postsynaptic currents (EPSCs) [28], and the NMDA antagonists upregulated the *SAP97 *mRNA expression in the cerebral cortex [14]. A more recent *in vitro *study using transfection, viral infection, small interference RNA, and antisense oligonucleotide techniques has demonstrated in the rat prefrontal cortex a functional link of the SAP97 proteins with the D4 type dopamine receptor that is possibly aberrant in mental illnesses including schizophrenia [29]. Taken together, the presumed dysregulation of the *SAP97 *gene connected to the rs9843659 polymorphism could lead to the plausible NMDA receptor deficits and/or abnormal dopamine neurotransmission in patients with schizophrenia.

The replicated male-selective genetic association of *SAP97 *gene with schizophrenia appears to be in line with the data indicating the sex-specific genetic associations with the disorder reported in several genes such as Disrupted-In-Schizophrenia 1 (*DISC1*), reelin (*RELN*) and D-amino acid oxidase (*DAO*) [30-32]. These phenomena might be related to the well-known gender differences in schizophrenia [33] that, for instance, include the clinical observations that male patients tend to exhibit an earlier onset and severer course than female patients, although the genetic basis of the sex-differences in schizophrenia remains unclear. Consequently, the male-specific association seen in the *SAP97 *gene could produce different influences on the putative disturbed neurotransmission via the NMDA receptor between male and female patients with schizophrenia.

## Conclusions

In conclusion, the present findings have confirmed that the glutamatergic transmission-linked and developmentally-regulated schizophrenomimetic-responsive gene *SAP97 *is associated with male patients with schizophrenia. As this unique association could provide a clue to elucidating the pathological changes in the glutamate system and their gender differences, further investigations are required in the larger and non-Japanese populations to extend its validity.

## List of abbreviations

SAP97: synapse-associated protein 97; AMPA: L-alpha-amino-3-hydroxy-5-methyl-4-isoxazole propionic acid; NMDA: N-methyl-D-aspartate; SNP: single nucleotide polymorphism; PCP: phencyclidine; DSM-IV: Diagnostic and Statistical Manual of Mental Disorders, fourth edition; M.I.N.I.: Mini-International Neuropsychiatric Interview; HWE: Hardy-Weinberg equilibrium; GWAS: genome-wide association studies; CNV: copy-number variation; MDR1: Multidrug Resistance 1; EPSCs: excitatory postsynaptic currents; DISC1: Disrupted-In-Schizophrenia 1; DAO: D-amino acid oxidase.

## Competing interests

The authors declare that they have no competing interests.

## Authors' contributions

AU undertook the statistical analysis and wrote the manuscript. J-KS carried out the SNP genotyping and collected the data. NY supervised the data collection and analysis. YI and HK organized the recruitment of subjects and prepared them for the blood samples. TN designed and managed the entire procedure of this study. All authors contributed and have approved the final manuscript.
